# Arabidopsis RabF1 (ARA6) Is Involved in Salt Stress and Dark-Induced Senescence (DIS)

**DOI:** 10.3390/ijms18020309

**Published:** 2017-02-01

**Authors:** Congfei Yin, Sazzad Karim, Hongsheng Zhang, Henrik Aronsson

**Affiliations:** 1 State Key Laboratory of Crop Genetics and Germplasm Enhancement, Nanjing Agricultural University, Nanjing 210095, China; congfei_yin@163.com (C.Y.); hszhang@njau.edu.cn (H.Z.); 2 Department of Biological and Environmental Sciences, University of Gothenburg, Box 461, Gothenburg SE-40530, Sweden; karim.sazzad@gmail.com

**Keywords:** ARA6, chloroplast, RabF1, salt stress, senescence, vesicle

## Abstract

Arabidopsis small GTPase RabF1 (ARA6) functions in endosomal vesicle transport and may play a crucial role in recycling and degradation of molecules, thus involved in stress responses. Here we have reported that complementary overexpression lines RabF1^OE^ (overexpression), GTPase mutants RabF1^Q93L^ (constitutively active) and RabF1^S47N^ (dominant negative) lines show longer root growth than wild-type, *rabF1* knockout and *N*-myristoylation deletion (Δ1−29, N-terminus) complementary overexpression mutant plants under salt induced stress, which indicates that *N*-myristoylation of RabF1 is indispensable for salt tolerance. Moreover, *RabF1* is highly expressed during senescence and RabF1^OE^ lines were more tolerant of dark-induced senescence (DIS) than wild-type and *rabF1*.

## 1. Introduction

Small GTPase Rab proteins are mainly involved in membrane transport between organelles, through vesicle transport of cargo proteins to their destinations. They also play a role in activating effector proteins to regulate different stages of membrane transport within the organelles and promote the downstream application of other proteins for a variety of regulatory roles [1,2]. During vesicle transport, Rab GTPases regulate tethering and fusion of transport vesicles to target membranes by acting as a molecular switch, cycling between a GDP (guanosine diphosphate) bound inactive state and a GTP (guanosine triphosphate) bound active state. Rab proteins interact with other regulatory and effector proteins to regulate the cycle of GDP/GTP binding and GTP hydrolysis. They have regulatory roles in vesicle budding from the donor membrane followed by uncoating, movement through the cell, tethering and docking in the vicinity of the acceptor membrane and final delivery of cargo to the target membrane by membrane fusion, as well as cargo sorting during coated vesicle transport [3,4,5,6]. The diversity of the Rabs mirrors their various roles in the eukaryotic cell, including membrane and vesicle transport, growth and development, signalling pathways, defence mechanism and stress responses and [7,8]. Out of the 57 Rabs in Arabidopsis the RabF1 is one of the most unique Rab GTPases in higher plants and among the eukaryotic Rab families. RabF1 characterizes by its association to the membranes with its unique N-terminal myristoylation site, rather than the common but essential carboxy-terminal geranylgeranylation CC motif for all other Rab proteins to be prenylated (membrane association) [4,9].

In *Arabidopsis thaliana* RabF1 (ARA6) has been extensively studied for its regulatory parts in endosomal transport via the assembly of a distinct SNARE (soluble *N*-ethylmaleimide-sensitive factor attachment protein receptors) complex in [10,11,12]. In plant N-terminal myristoylation not only plays a crucial role in membrane targeting but modulates signal transduction in responses to environmental stress such as salt stress, growth regulation, disease resistance and endocytosis [13]. An Arabidopsis gene, *SOS3* (salt overly sensitive 3) which contains a consensus N-terminal myristoylation sequence showed functional role in salt tolerance to salt [14]. RabF1 has been reported as *N*-myristoylated protein in vivo and in vitro [10,15]. RabF1 may have a functional role in plants’ response to abiotic stress such as salinity and it has been suggested that it is closely associated with membranes and expressed constitutively [10,11]. RabF1 has two homolog proteins in Arabidopsis, RabF2a/Rha1 and RabF2b/Ara7, both localized in the prevacuolar compartment (PVC). However, AtRabF1 absolutely differs from these two proteins for its N-terminal myristoylation site. It has been suggested that RabF1 is localized to the endosomes and the plasma membrane but not to the trans-Golgi network (TGN), where it plays a regulatory role in the formation of a SNARE complex comprising endosome-residing R-SNARE, VAMP727 and a plasma membrane Q-SNARE SYP121 [11]. However, CaARA6—the RabF1 homologue in *Chara australis*—has been found to be localized at the plasma membrane, the TGN, and multivesicular endosomes (MVEs), suggesting involvement in endosomal transport [16]. Moreover, it has been suggested that a guanine nucleotide exchange factor (GEF) VPS9a is the activator for RabF1 to conform the GTP-bound form’s in vitro structures [17]. Overexpression of constitutively active *AtRabF2b* suppresses abnormal phenotypes of *atvps9a-2*. In contrast, the overexpression of *AtRabF1* with an equivalent mutation does not suppress abnormal phenotypes of *atvps9a-2*. *AtRabF1* plays a specific role in transport from endosomes to the plasma membrane, while conventional Rab5 proteins, *AtRabF2a* and *AtRabF2b* mainly work in transport to vacuoles through endosomes [10,11,12,18]. Recently, it has also been suggested that *AtRabF1* is responsible for starch and sugar homeostasis through the function of Qua-Quine Stach gene (*QQS*), and the proliferation of *Pseudomonas syringae* pv. tomato DC3000 was repressed in *AtRabF1* knockout mutant [19].

However, the complete picture of the function of RabF1 is not yet fully understood, although several recent works on RabF1 have highlighted the unique properties of the protein [10,11,19]. We report that Arabidopsis RabF1 plays an important role in salt-induced stress, which is linked to its *N*-myristoylation site. Moreover, RabF1 is highly expressed during senescence, thus showed its protective effect in dark-induced senescence (DIS) leaves and photosynthetic parameter such as chlorophyll content.

## 2. Results and Discussion

### 2.1. RabF1^OE^, RabF1^Q93L^ and RabF1^S47N^ Lines Are More Tolerant to Salt Stress Compared to Wild-Type, rabF1 and RabF1^Δ1^^−29^

**Figure 1:**
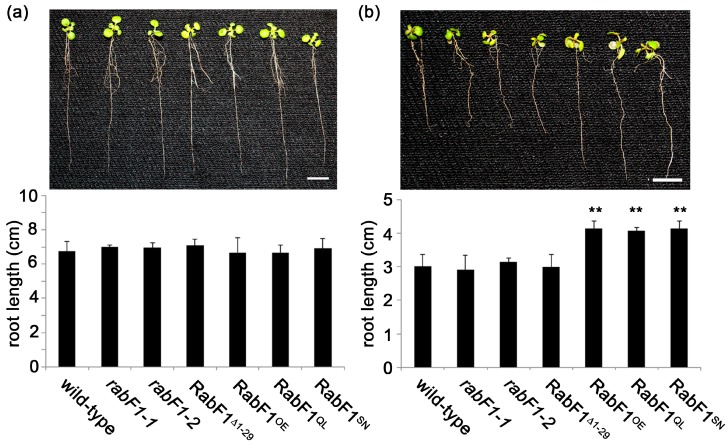
RabF1 overexpression lines RabF1^OE^, RabF1^Q93L^ and RabF1^S47N^ are tolerant to salt stress compared to wild-type, *rabF1* and RabF1^Δ1−29^. Root lengths of plants (**a**) without or (**b**) with salt stress with graphical representation of the results below. Error bars represent ± SE (*n* ≥ 20). Asterisks indicate values that are significant different from wild-type (** *p* ≤ 0.01). Bars = 1 cm.

Two knockout mutants for *RabF1*, *rabF1-1* and *rabF1-2*, were collected from NASC for genotypic and phenotypic analysis (Figure S1a). PCR with T-DNA-based primers showed that both *rabF1-1* and *rabF1-2* had T-DNA insertions disrupting the *RabF1* gene and were homozygous for this mutation (Figure S1b,c). RT-PCR confirmed the absence of expression of *RabF1* in both mutant lines (Figure S1d), and no RabF1 protein was detected in total leaf extract of the mutant lines after immunoblotting using the antibody against RabF1 (Figure S1e). Transgenic complementary lines in *rabF1-1* mutant background with overexpressing RabF1-EYFP (RabF1^OE^), constitutively active 35S-RabF1^Q93L^-EYFP (RabF1^Q93L^), dominant negative 35S-RabF1^S47N^-EYFP (RabF1^S47N^) and 35S-RabF1^Δ1−29^-EYFP (RabF1^Δ1−29^) plants were used for salt stress. Expression level was checked by RT-PCR for two lines of each construct (Figure S2) but only one representative line of each construct is shown in figures. According to the Arabidopsis eFP Browser (http://bar.utoronto.ca/efp/cgi-bin/efpWeb.cgi), *RabF1* responds to osmotic stress, and recently, it was reported that RabF1^Q93L^ lines were more tolerant to salt stress [11,12]. Therefore, we grew RabF1^OE^, RabF1^Q93L^, RabF1^S47N^ and RabF1^Δ1−29^ overexpression lines under conditions of 100 mM NaCl salt stress. Without added salt all lines exhibited similar root lengths i.e., no differences were observed (Figure 1a). During salt stress the overexpression lines (RabF1^OE^, RabF1^Q93L^ and RabF1^S47N^) showed significantly longer root lengths (Figure 1b) compared to that of wild-type, *rabF1* and the overexpression line RabF1^Δ1−29^, which indicated that the RabF1 has a positive effect over salt stress. Compared with wild-type roots, *RabF1^OE^*, *RabF1^Q93L^* and *RabF1^S47N^* overexpression lines had roots that were, respectively, 37.1%, 35.1% and 37.1% longer when exposed to salt stress (Figure 1b). In addition, the roots of *rabF1* were of similar length under salt stress conditions as the wild-type, indicating that knockout of *RabF1* had no effect on root growth in saline conditions (Figure 1b).

In this study, both the RabF1^Q93L^ and RabF1^S47N^ lines exhibited similar patterns of salt tolerance despite being different in the conformation of their GTPase domain. It was found that that all the overexpression lines except *RabF1^Δ1−29^* were more tolerant to salt stress condition than wild-type and *rabF1* lines, indicated by significantly longer roots (Figure 1b). The roots of *RabF1^Δ1−29^* were similar to the root length of wild-type and *rabF1*. Thus, we concluded that it was not the GTPase domain (active/inactive states) of RabF1 that generated the root phenotypic effect on salt stress condition. Therefore, it is more likely that the *N*-myristoylation ability of RabF1 is responsible for its salt stress related phenotype, as it was observed in the salt tolerance gene *SOS3* (salt overly sensitive 3) where salt tolerance is dependent on *N*-myristoylation [14].

### 2.2. RabF1 Is Involved in Leaf Senescence

**Figure 2:**
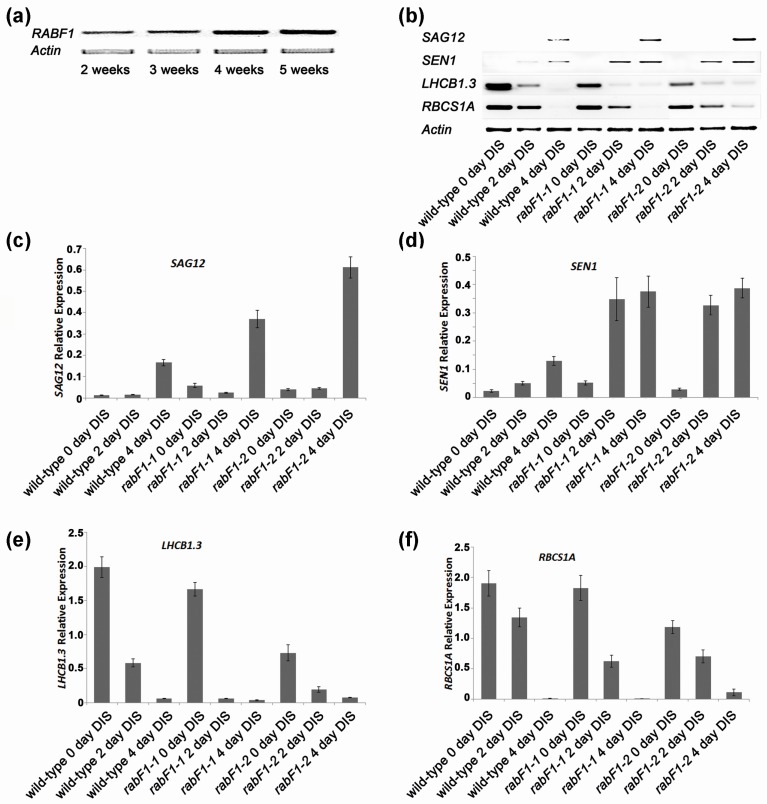
The expression of *RabF1*. (**a**) RT-PCR results show that the expression of *RabF1* in different stages of Arabidopsis is upregulated with aging (in weeks); (**b**) Senescence-related marker gene expression in dark induced plants of the wild-type and *rabF1* lines. RT-PCR results revealed the expression of senescence markers *SAG12*, *SEN1*, *LHCB1.3* and *RBCS1A.* Expression of *Actin* was used as a control to demonstrate the equal amount of mRNA per sample used as a template. Relative expression versus actin was estimated for (**c**) *SAG12*, (**d**) *SEN1*; (**e**) *LHCB1.3* and (**f**) *RBCS1A* Three biological and four technical replicates were used for each data collected.

Interestingly, salt stress promotes leaf senescence, thus improving salt tolerance delays senescence [20]. However, there is little information about the involvement of Rab proteins in senescence-related processes. Recently, up-regulation of senescence-related genes has been reported under DIS in rice *OsRab7B3* overexpression line. During the normal senescence process, over-expressing *OsRab7B3* transgenic plants had yellowing leaves earlier than wild-type plants. Thus, it was suggested that *OsRab7B3* is a positive factor, which promotes senescence in rice [21]. Knowing this we wanted to determine if RabF1 had a role in senescence. We examined the expression of the *RabF1* gene using a publicly available microarray database and Genevestigator v3 [22,23]. Expression data from all high-quality ATH1 (22 k) arrays were analysed [24]. The developmental expression analysis revealed that *RabF1* has a high expression level throughout all tissues, with a slightly higher level in senescing tissues. In senescing tissue, the expression value is ca. 14 Units, whereas in other tissues it is ca. 13 Units (Figure S3). The expression of *RabF1* at the different developmental stages was verified by using leaves from two- to five-week-old plants. RabF1 was more highly expressed in four- and five-week-old leaves than in younger leaves (Figure 2a). Thus, the expression of *RabF1* increased with the ageing of plants. Light is very important during plants’ growth and development phases. Light levels outside the optimal range will accelerate plant senescence. Complete darkness represents extreme conditions that will accelerate the process of senescence. As senescence progresses, there is a sequence of physiological activities that change as a result of catabolic processes such as, chlorophyll degradation, decline in the total amount of mRNA and protein in the leaf tissues, devitalization of photosynthesis elements and cell lysis.

Over recent decades several genes have been characterized with respect to their roles in natural and induced senescence studies. Some of them are used as good marker genes for senescence, for example; *SAG12* (senescence-associated gene 12) exhibits increased expression throughout the progression of senescence and is highest at the final stage of senescence; *SEN1* (senescence-associated protein1) expression has been found to be highest at the beginning of DIS and to be responsive to plant defence signals in Arabidopsis; *RBCS1A* (a member of the Rubisco small subunit of genes) is associated with accumulation of Rubisco in Arabidopsis leaves and works additively with other Rubisco small subunit genes to yield sufficient Rubisco for photosynthesis and is expressed in leaf tissues throughout the developmental period, but is generally down-regulated in old plants; and *LHCB1.3* (*CAB1*) a subunit of the light-harvesting complex II (LHCII) of Arabidopsis is generally expressed constitutively [25,26,27,28,29,30,31,32].

Under DIS both senescence marker genes, *SAG12* and *SEN1*, were highly expressed in leaves from *rabF1* mutants compared to the wild-type plants (Figure 2b–d). This indicates that RabF1 could have a negative regulative role of these genes. However, the expression of *RBCS1A*, and *LHCB1.3* were in general down-regulated in four-week-old ageing *rabF1* plants more rapidly than wild-type plants exposed to the dark treatment (Figure 2b,e,f) not only indicating that the dark treatment worked (green tissues and leaves are affected by DIS negatively) but also indicating that RabF1 could affect these genes in a slightly positive manner when present. Thus, the absence of RabF1 indicates a more rapid senescence in mutants than wild-type plants, and so RabF1 was identified as being involved in senescence-related processes.

### 2.3. RabF1 Plays an Important Role in Dark-Induced Senescence (DIS)

**Figure 3:**
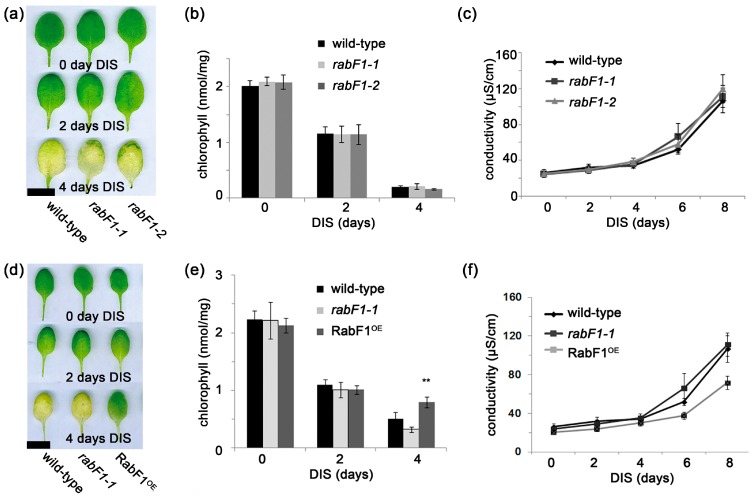
Effect of DIS, chlorophyll content and conductivity among the RabF1^OE^ overexpression lines, wild-type and *rabF1*. (**a**–**c**) The wild-type and *rabF1* lines responded similarly during DIS. No significant difference in chlorophyll content or conductivity was observed between wild-type and *rabF1* plants during DIS treatment. Compared to the wild-type and *rabF1* lines, the overexpression mutant (**d**–**f**) RabF1^OE^ line was found to be more tolerant to DIS and had significantly higher chlorophyll content after four days of DIS treatment and lower conductivity after eight days of DIS treatment. The experiments were repeated three times and got similar trends. Error bars represent ± SE (*n* ≥ 5). Asterisks indicate values that are significant different from wild-type (** *p* ≤ 0.01). Bars (**a**, **d**) = 1 cm.

To determine the role of RabF1 in DIS, the 4th leaves of four-week-old wild-type and *rabF1* plants were detached and incubated in a dark chamber for 2–4 days. After the DIS, no obvious phenotypic difference was observed between samples from wild-type and *rabF1* plants. Moreover, no significant difference was found for chlorophyll content or electric conductance [33] measurements taken to quantify cell damage at different time points (Figure 3a–c).

However, *RabF1^OE^* lines were compared with wild-type and *rabF1-1* under DIS. A visible difference was observed after 4 days of DIS, when *RabF1^OE^* lines had green leaves while wild-type and *rabF1* showed evidence of senescence in the form of bleached leaves (Figure 3d–f). This indicates that RabF1^OE^ lines were more tolerant to DIS. Interestingly, the chlorophyll content at 4 days of DIS correlated with the greenness of leaves at the same stage (Figure 3d–e), indicating that RabF1 is involved in the mechanism protecting against chlorophyll degradation. Thus, *RabF1^OE^* lines maintained higher chlorophyll content after 4 days of DIS compared to wild-type and *rabF1* (Figure 3e). Conductivity at different time points was also measured and RabF1^OE^ lines had lower conductivity (Figure 3f). All these results revealed that RabF1^OE^ lines were more tolerant to DIS than wild-type and *rabF1* mutant lines. Therefore, RabF1 could be a negative regulator in Arabidopsis during DIS. The data shows that even if the absence of RabF1 had a slightly negative effect on *RBCS1A,* and *LHCB1.3* (Figure 2b,d,e) this was not enough to distinguish any clear phenotype linked to green tissue or leaves between *rabF1* and wild-type plants during DIS (Figure 3). However, when *RabF1* being overexpressed it shows a protective phenotype (Figure 3) in accordance with the suggestion to be a negative regulator for *SAG12* and *SEN1* (Figure 2b–d). In this study we did not focus on the RabF1 GTPase states or *N*-myristoylation for the senescence. This can be addressed in a future elaborative study using existing mutant lines.

### 2.4. Conclusions

The data reported here showed that RabF1 plays a role in recycling and degradation processes during senescence and stress responses. Salt stress data from overexpression RabF1 lines without the *N*-myristoylation site strongly indicate that membrane anchoring plays an important role in the increased tolerance to salt stress (Figure 1). The response to salt stress might be linked to close association of RabF1 to e.g., the plasma membrane, since it contains several transporters involved in signaling in response to salt stress [34]. Thus, the exact mechanism behind the involvement of the RabF1 *N*-myristoylation to salt stress still needs to be further investigated.

For DIS related senescence, RabF1 as a GTPase might have a protective role against senescence by protecting the photosynthetic machinery, which is plausible considering the observations in Figure 3d,e. Whether RabF1 has direct role with chloroplasts, as suggested by bioinformatics studies [35], remains to be elucidated. If RabF1 is considered to be in an active state in the wild-type and overexpressed lines when performing the DIS assays (in contrast to knockout mutants where *RabF1* is not present), the RabF1 GTPase function can be linked to the senescence phenotype (Figure 3). The *N*-myristoylation should not be responsible for the DIS related phenotype as the wild-type and overexpressed lines both contain *N*-myristoylation sites but did not show similar leaf senescence pattern during DIS experiments (Figure 3). Still additional future experimental data are needed to resolve the exact role of the GTPase for senescence.

## 3. Materials and Methods

### 3.1. Plant Material

All *Arabidopsis* lines were grown as previously described [35,36] unless otherwise mentioned for specific experiments. The T-DNA insertion mutants (*rabF1*) for *ARA6/RabF1* (At3g54840), SAIL_98_E08 (*rabF1-1*) and WiscDsLox481-484C9 (*rabF1-2*) were obtained from NASC (http://arabidopsis.info/). The background line for all of these mutant lines, ecotype Col-0 (wild type, WT), was used as the control for all experiments. The *rabF1-1* T-DNA band was detected by LB1 (5′-GCCTTTTCAGAAATGGATAAATAGCCTTGCTTCC-3′) and atrabF1-1RP (5′-AACGAGGCTCCAACAGTTACC-3′), while the *RabF1* gene was detected by atrabF1-1LP (5′-TTGGAGAAACCGAATTGATTG-3′) and atrabF1-1RP (5′-AACGAGGCTCCAACAGTTACC-3′, Table S1). The *rabF1-2* T-DNA band was detected by P745 (5′-AACGTCCGCAATGTGTTATTAAGTTGTC-3′) and atrabF1-2RP (5′-TTCACTCACATCAGAGCATGG-3′), while the *RabF1* gene was detected by atrabF1-2LP (5′-TTTCCGAAGGTGTAATCATCG-3′) and atrabF1-2RP (5′-TTCACTCACATCAGAGCATGG-3′, Table S1).

### 3.2. Total RNA Isolation, RT-PCR and Semi-Quantitative RT-PCR

An RNeasy plant mini-kit (Qiagen AB Sweden) was used to purify total RNA with an in-column DNase treatment. One microgram of total RNA/sample was used as a template while performing RT-PCR using an illustra Ready-To-Go RT-PCR Beads (0.5 mL tubes) kit (GE Healthcare Sverige AB, Sweden); the RT-PCR procedure was performed according to the manufacturer’s instructions. For semi-quantitative reverse transcription-PCR, two mg of total RNA was used for the synthesis of the first-strand of cDNA with a RevertAid™ H Minus First Strand cDNA Synthesis Kit (Life Technologies Europe BV, Sweden) with oligo(dT)18. PCR cycle was terminated after 25 cycles for *Actin*, 24 cycles for *RBCS1A*, 27 cycles for *LHCB1.3* (*CAB1*), 26 cycles for *SEN1* (senescence-associated protein 1), and 27 cycles for *SAG12* (senescence-associated gene 12) genes. Specific primers to respective genes were as follows: Actin (forward 5′-AGAGATTCAGATGCCCAGAAGTCTTGTT-3′, and reverse 5′-AACGATTCCTGGACCTGCCTCATC-3′); *RBCS1A* (forward 5′-CCACCCGCAAGGCTAACAAC-3′, and reverse 5′-TTCGGAATCGGTAAGGTCAGG-3′); *LHCB1.3* (forward 5′-CCAGAGGCATTCGCTGAGTTG-3′, and reverse 5′-CCTTACCAGTGACGATGGCTTG-3′); *SEN1* (forward 5′-GTCATCGGCTATTTCTCCACCT-3′, and reverse 5′-GTTGTCGTTGCTTTCCTCCATC-3′); *SAG12* (forward 5′-CAGCTGCGGATGTTGTTG-3′, and reverse 5′-CCACTTTCTCCCCATTTTG-3′, Table S1). Three biological and four technical replicates were used for the semi-quantitative RT-PCR, and relative expression was estimated using Image J 1.46r (Available online: http://rsb.info.nih.gov/ij/).

### 3.3. RabF1 Cloning

The *RabF1* coding sequence, corresponding to the Arabidopsis Genome Initiative (AGI) accession number At3g54840, was cloned with Gateway^TM^ technology (Invitrogen, http://www.invitrogen.com). The sequence was amplified by PCR from a complete cDNA clone (Gene bank accession no. BT002860). The PCR fragment was inserted into the pDONR^TM^ vector and transferred into destination vectors (forward 5′-CACCATGGGATGTGCTTCTTCTCTT-3′, and reverse 5′-TGACGAAGGAGCAGGACGA-3′, Table S1). For constitutively active (locked in GTP-bound state) mutants of RabF1 (Q93L) and dominant negative (locked in GDP-bound state) mutants of RabF1 (S47N), each mutation was introduced by PCR-based mutagenesis into the cDNA sequence as well as inserted into the vector for the subsequent construction of YFP (yellow fluorescence protein) fluorescently tagged proteins. The PCR-based mutagenesis primers were as follows: RabF1 (Q93L) (forward 5′-TGGGATACAGCAGGACTGGAGAGGTATTAAACC-3′, and reverse 5′-GGTTTAATACCTCTCCAGTCCTGCTGTATCCCA-3′); RabF1 (S47N) (forward 5′-TCTGGTGTTGGTAAAAATTGTATTGTCC-3′, and reverse 5′-GGACAATACAATTTTTACCAACACCAGA-3′, Table S1). For the myristoylation mutant (Δ1−29) amplification by PCR was performed ignoring the first 29 amino acids of RabF1 to remove the myristoylation site (forward 5′-CACCATGGGTCAGTTTGACGCTACA-3′, and reverse 5′-TGACGAAGGAGCAGGACGA-3′, Table S1). The fragment was inserted into the vector as above.

### 3.4. Expression of 35S::RabF1-EYFP (RabF1^OE^), 35S::RabF1^Q93L^-EYFP (RabF1^Q93L^), 35S::RabF1^S47N^-EYFP (RabF1^S47N^), 35S::RabF1^Δ1−29^-EYFP (RabF1^Δ1−29^) in rabF1

*RabF1* cDNA was transferred into the binary vector pH7FWG2/pH7YWG2 [37] to express the 35S::RabF1-YFP fusion protein. These vectors, containing the hygromycin resistance gene, were used to transform *Agrobacterium tumefaciens* strain CV3101 by the heat-shock method [38]. Stable transformation of Arabidopsis with CV3101 Agrobacteria was achieved using the floral-dip method [39]. Transgenic plants were selected on MS medium supplemented with hygromycin B (15 ug·mL^−1^). Two lines of each construct were used (Figure S2) but only one are shown as representative in figures. Positive transgenic plants were confirmed by PCR assays. The specific primers used were for RabF1^OE^, RabF1^Q93L^, and RabF1^S47N^ (forward 5′-CACCATGGGATGTGCTTCTTCTCTT-3′, and reverse 5′-GCGAAGCACTGCAGGCCGTAGCCGAA-3′); and for RabF1^Δ1−29^ (forward 5′-CACCATGGGTCAGTTTGACGCTACA-3′, and reverse 5′-GCGAAGCACTGCAGGCCGTAGCCGAA-3′, Table S1).

### 3.5. Plant Growth for Salt Stress

Plants were grown on MS plates (0.5% sucrose) for four days. Four-day-old seedlings were moved to vertical plates containing 100 mM NaCl, and the plates were transferred into long day conditions for another six days. Ten-day-old seedlings were used to measure root length. The root lengths of seedlings were measured and analysed using Image J 1.46r (Available online: http://rsb.info.nih.gov/ij/).

### 3.6. Dark-Induced Senescence (DIS), Chlorophyll and Conductivity Measurements

For the DIS treatment, the 4th leaf from four-week-old plants grown under short day conditions was detached and placed in a Petri dish covered with sterilized filter paper containing 10 mL of 3 mM MES (pH 5.7). The leaf was placed with its adaxial side facing up and incubated in a dark chamber at 22 °C for the indicated time. The chlorophyll was determined photometrically as previously described after DIS treatment [40,41]. The mean and standard deviation were calculated for each data set where appropriate. For each ion leakage measurement, six 0.6 cm diameter discs were punched from a fully expended rosette leaf from plants just prior to blotting. Three leaves were sampled per plant. The discs were placed into 6 mL distilled water and incubated in a dark chamber at 22 °C. Conductance was measured as previously described [33,42] at the desired time points and the mean and standard deviation calculated accordingly.

## Supplementary Materials

Supplementary materials can be found at www.mdpi.com/1422-0067/18/2/309/s1.
