# Supplementary material for: Arabidopsis RabF1 (ARA6) Is Involved in Salt Stress and Dark-Induced Senescence (DIS)
Source: Int J Mol Sci. 2017 Feb 1;18(2):309. doi: 10.3390/ijms18020309 (PMC5343845; doi:10.3390/ijms18020309)
Supplement: Supplementary file 1 [file ijms-18-00309-s001.docx]

Supplementary Material:
Arabidopsis RabF1 (ARA6) Is Involved in Salt Stress and Dark-Induced Senescence (DIS)

Congfei Yin, Sazzad Karim, Hongsheng Zhang and Henrik Aronsson


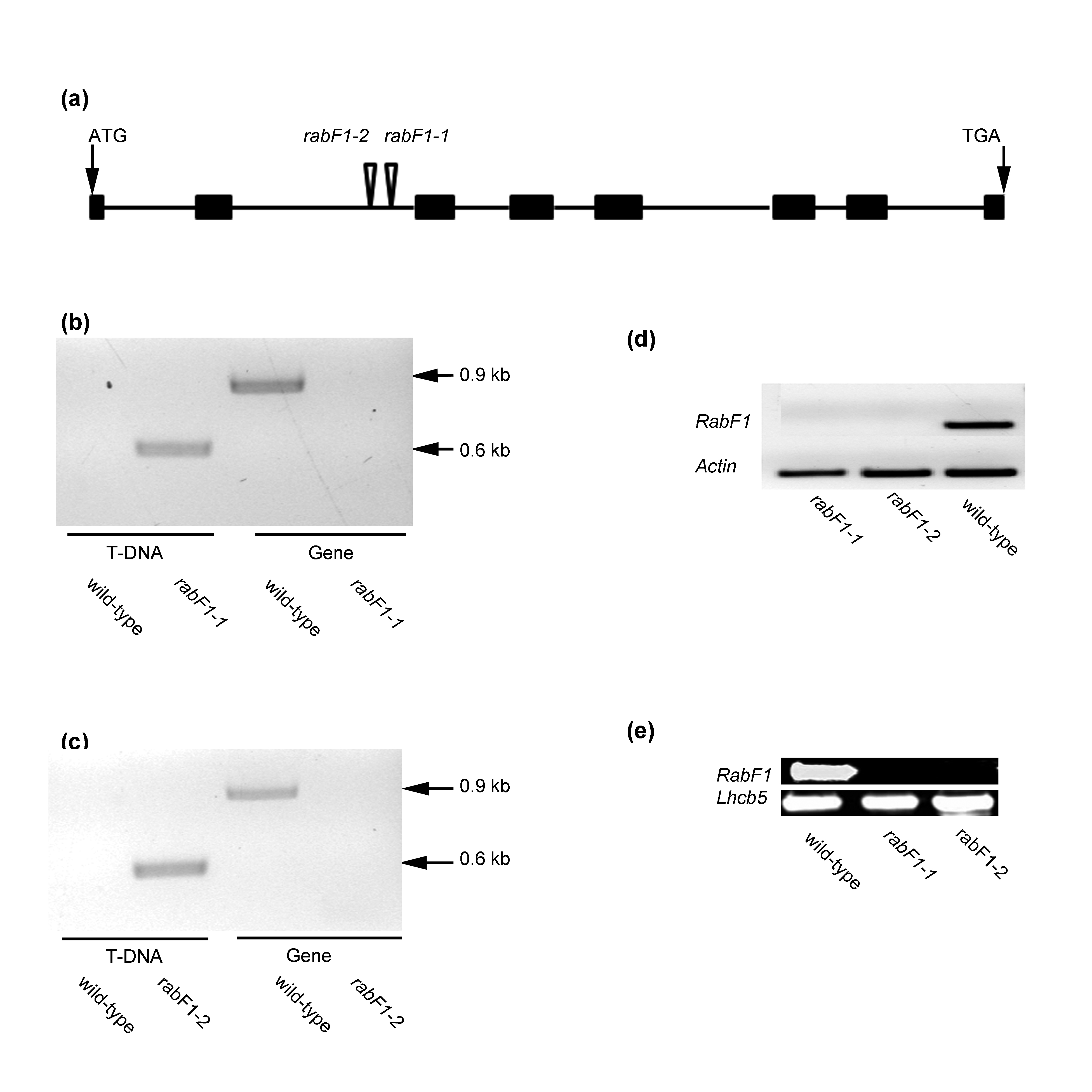


**Figure S1.** Verification of two knockout lines of *RabF1*. (**a**) Two *Arabidopsis* T-DNA insertion mutants of *RabF1,* *rabF1-1* and *rabF1-2* were collected with Col-0 as the wild-type background from NASC. The diagram shows the T-DNA insertion sites (triangles) of mutants to create the knockout mutants. Black boxes represent exons of *RabF1*; (**b**–**c**) PCR analysis revealed single PCR fragments from *rabF1-1* and *rabF1-2* genomic DNA when using the *RabF1* gene and inserted T-DNA-specific primers (T-DNA) but no fragments from upstream or downstream of the *RabF1* gene-specific primers (Gene), suggesting a homozygous condition in both lines for their knockout insertion. The corresponding fragment from the *RabF1*-related primers in wild-type genomic DNA was used as a control; (**d**) RT-PCR analysis showed *rabF1-1* and *rabF1-2* lacking the expression of *RabF1* while the wild-type expressed *RabF1*. *Actin* was detected in similar amounts in each lane; this was used as a control for equal loading; (**e**) RabF1 is detected in the total leaf extract of wild-type plants but not in *rabF1* knockout mutants (*rabF1-1* and *rabF1-2*). Western blot was performed using a RabF1-specific antibody and the expected size of full RabF1 22 kDa, was detected. Detection of RabF1 was performed in 5 µg of total leaf extract per lane for all the lanes.


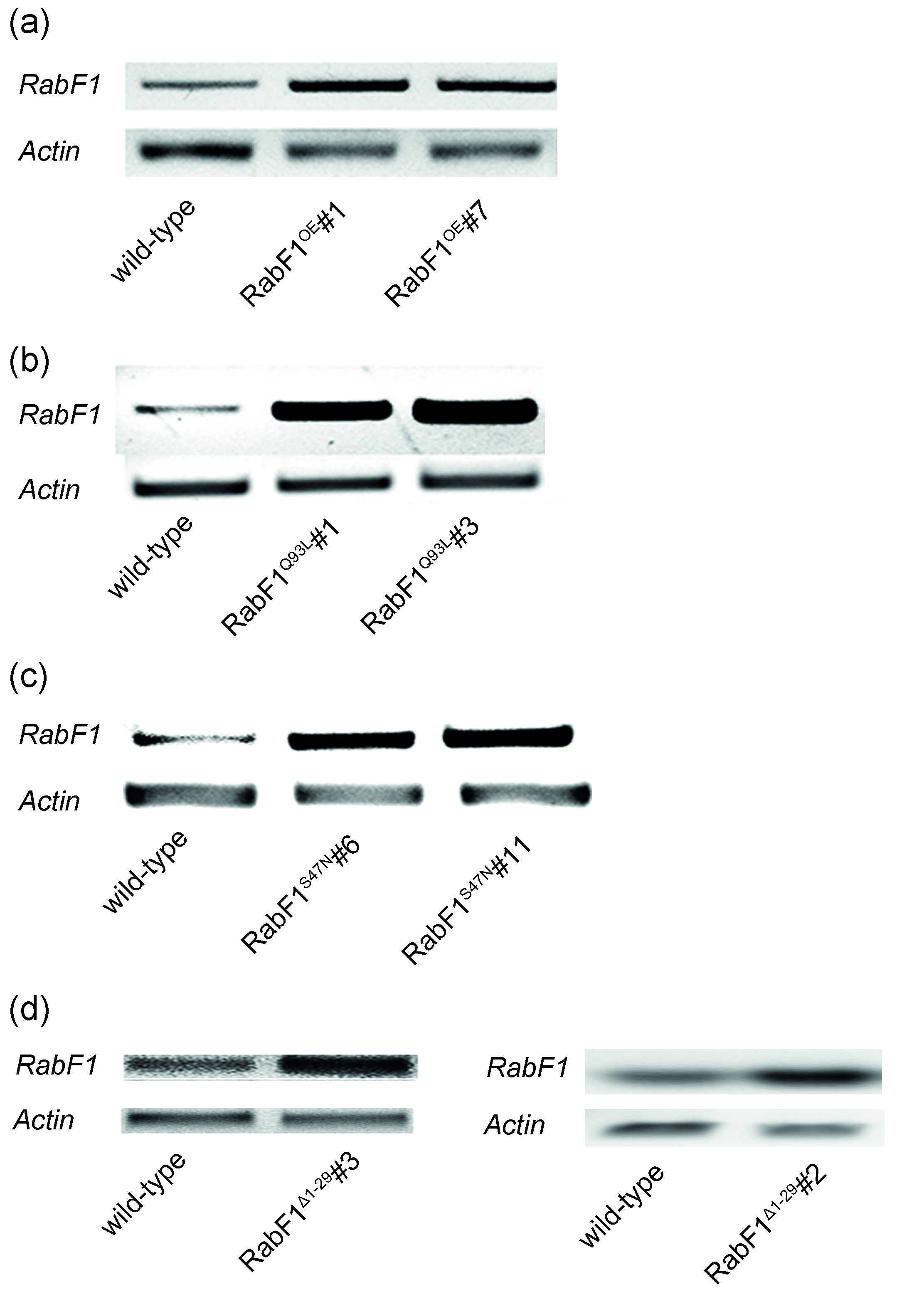


**Figure S2.** Different complementary transgenic lines overexpress *RabF1*. Expression of *RabF1* in different transgenic lines overexpressing *RabF1* compared to the wild-type. (**a**) RabF1^OE^ (35S-RabF1-EYFP) lines overexpressing *RabF1* without any change; (**b**) Constitutively active RabF1^Q93L^ (35S-RabF1^Q93L^-EYFP) lines overexpressing *RabF1*; (**c**) Dominant negative RabF1^S47N^ (35S-RabF1^S47N^-EYFP) lines overexpressing *RabF1*. (d) Myristoylation RabF1^Δ1–29^ (35S-RabF1^Δ1–29^-EYFP) lines overexpressing *RabF1*.


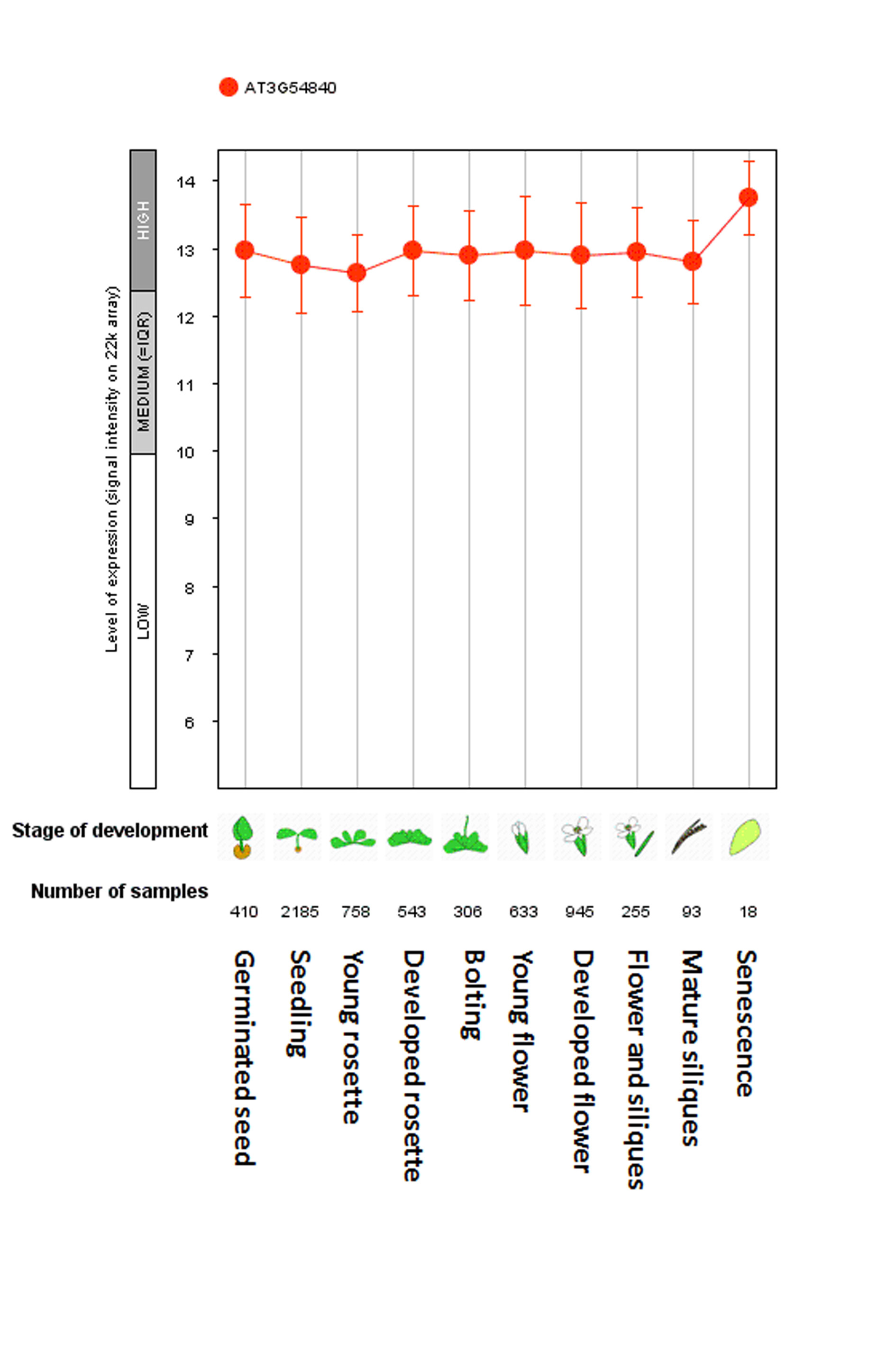


**Figure S3.** Expression of *RabF1* at different developmental stages of Arabidopsis. Developmental expression profile of *RabF1* according to the publicly available Affymetrix GeneChip microarray data. Data were retrieved using the Genevestigator v3 analysis tool (www.genevestigator.com/gv/plant.jsp) and prepared with the Meta-Profile Analysis tool using development representations in scatter-plot format. Data from all high-quality ATH1 (22 k) arrays were analysed [24]. The plot shows that *RabF1* has a high expression level throughout all tissues with the highest level in senescing tissues.

**Table S1.** Primers used for knockout mutant identification, construct, cloning and RT-PCR.

| **Name** | **5′–3′** | **Function** |
| --- | --- | --- |
| LB1 | GCCTTTTCAGAAATGGATAAATAGCCTTGCTTCC | knockout mutant identification |
| atrabF1-1LP | TTGGAGAAACCGAATTGATTG |  |
| atrabF1-1RP | AACGAGGCTCCAACAGTTACC |  |
| P745 | AACGTCCGCAATGTGTTATTAAGTTGTC |  |
| atrabF1-2LP | TTTCCGAAGGTGTAATCATCG |  |
| atrabF1-2RP | TTCACTCACATCAGAGCATGG |  |
| EGFP Rev | CTGAAGCACTGCACGCCGTAGGTCAG | construct |
| EYFP Rev | GCGAAGCACTGCAGGCCGTAGCCGAA |  |
| AtRabF1^∆1–29^For | CACCATGGGTCAGTTTGACGCTACA | cloning |
| AtRabF1For | CACCATGGGATGTGCTTCTTCTCTT |  |
| AtRabF1Rev | TGACGAAGGAGCAGGACGA |  |
| RabF1^QL^ For | TGGGATACAGCAGGACTGGAGAGGTATTAAACC |  |
| RabF1^QL^ Rev | GGTTTAATACCTCTCCAGTCCTGCTGTATCCCA |  |
| RabF1^SN^ For | TCTGGTGTTGGTAAAAATTGTATTGTCC |  |
| RabF1^SN^ Rev | GGACAATACAATTTTTACCAACACCAGA |  |
| AtRabF1For | ATGGGATGTGCTTCTTCTCTT | RT-PCR |
| AtRabF1Rev | AGCAGAATACCTCTCCTGTCC |  |
| ActinFor | AGAGATTCAGATGCCCAGAAGTCTTGTT |  |
| ActinRev | AACGATTCCTGGACCTGCCTCATC |  |
| SEN1 For | GTCATCGGCTATTTCTCCACCT |  |
| SEN1 Rev | GTTGTCGTTGCTTTCCTCCATC |  |
| LHCB1.3 For | CCAGAGGCATTCGCTGAGTTG |  |
| LHCB1.3 Rev | CCTTACCAGTGACGATGGCTTG |  |
| RBCS1A For | CCACCCGCAAGGCTAACAAC |  |
| RBCS1A Rev | TTCGGAATCGGTAAGGTCAGG |  |
| SAG12 For | CAGCTGCGGATGTTGTTG |  |
| SAG12 Rev | CCACTTTCTCCCCATTTTG |  |
